# The Spirit of Adventure: A Driver of Attractiveness of the Hospitality Industry for Young People during a Pandemic Crisis

**DOI:** 10.3390/ijerph19041913

**Published:** 2022-02-09

**Authors:** Adriana Burlea-Schiopoiu, Mara Del Baldo, Samuel O. Idowu

**Affiliations:** 1 Department of Management, Marketing, Business Administration, University of Craiova, 200585 Craiova, Romania; 2 Department of Economics, Society and Politics, University of Urbino Carlo Bo, 61029 Urbino, Italy; mara.delbaldo@uniurb.it; 3 Guildhall School of Business and Law, London Metropolitan University, London EC2M 6SQ, UK; s.idowu@londonmet.ac.uk

**Keywords:** attractiveness, COVID-19, hospitality industry, job insecurity, perceptions, spirit of adventure, young people

## Abstract

The COVID-19 pandemic has strongly affected tourism and leisure activities worldwide, especially in the hospitality and tourism sectors. Within this context, this study aimed to evaluate the impact of the pandemic on the future attractiveness of the hospitality industry (HI) to young people. The conceptual model underpinning the empirical research proposes a direct relationship between job attractiveness and the spirit of youth adventure. Findings prove that young people are enthusiastic about working in the HI because they can easily practice their creative ideas. Communicating with other people and dealing with clients’ complaints is challenging for them in the pandemic crisis created by COVID-19. The results are of interest to policymakers in terms of suggestions on how to transform the challenges into opportunities by using the constraints imposed by the pandemic crisis that have limited the socialisation between people, enhancing the creativity of young people, and motivating them to work in the HI. Moreover, our research provides managers and other decisionmakers with some motivational factors to increase the attractiveness of their companies to young people and suggestions helpful to scholars involved in HI research to cultivate resilience capabilities by giving them inherent skills.

## 1. Introduction

In late 2019, our world was besieged by an unprecedented pandemic that has caused severe devastation to people’s lives, the global economy, the social and political environments globally [1]. It was reported that about 50 million people had lost their lives during that pandemic [2]. Governments of all nations are doing their utmost to support corporate bodies and individual citizens. While nation-states have begun reopening their economies, the surge in infections (i.e., the second and third waves) has raised calls for modified quarantine policies. However, the pandemic continues to plague all sectors of the global economy [3] and generate uncertainty.

The hospitality industry (HI) employs over 200 million people across the globe [4]; economically, it is a source of many jobs and supports livelihoods of millions of workers and their families. Moreover, it plays a crucial role in societalisation that the current pandemic has severely compromised, pointing out how extreme disruptive events affect the role of business in society [5].

Travel bans and border closure policies and restrictions have significantly disrupted tourism and leisure activities around the globe [6]. Strategies to flatten the COVID-19 curve (i.e., community lockdowns, social distancing, stay-at-home orders, travel and mobility restrictions) have resulted in many forced widespread requirements due to the risk of infection and death among the vulnerable community segments [7,8].

Consequently, these strategies significantly decreased the demand for hospitality businesses that were allowed to continue to operate [9].

The negative impacts of these actions have threatened the survival of many businesses, making employees redundant in large numbers. Top groups, such as Hilton and Marriot, have announced furloughs and Southwest Airlines asked its labour unions to accept pay cuts to avoid layoffs [10]. The World Travel and Tourism Council estimates that globally, somewhere between 98 to almost 200 million travel and tourism jobs are in jeopardy worldwide [11].

As a result of this extraordinary scenario, a growing number of researchers have turned attention to the implications for the future of tourism [12,13] and tourist destinations [14]. The behaviours of the HI operators are related to their critical concerns regarding the changes necessary to tackle the pandemic outbreaks and prepare for the next normality.

Taking into consideration the objective of our research to evaluate the impact of the pandemic crisis generated by COVID-19 on the future attractiveness of the hospitality industry (HI) to young people, we organized the paper as follows: Section 2 explores the literature on the HI in the COVID-19 scenario and introduces our hypotheses; Section 3 describes the methodology employed to present the theoretical model of the research, followed by data analysis and validation. The findings and the emerging issues are explored in Section 4. Section 5 closes the paper with our conclusions.

## 2. Theoretical Background and Hypotheses Development

In the recent years, several works have triggered an intense and ongoing debate on the HI in the face of the current global pandemic. A systemic review of the prior studies pointed out the most relevant topics and the methodologies used to investigate the impact of COVID-19 on the HI [15]. The studies mainly identified the implications relative to the recovery of the HI market demands, discussing the resumption of activities during and after the pandemic, the revenue losses, and the COVID-19 spread patterns in the HI, including event, cruise, hotel, and restaurant industries. The issues related to travel behaviours, preferences of customers, and social costs of the pandemic received less attention than several types of research that have deepened the understanding of implications of job losses and employment of hospitality workforce, safety, and health aspects.

### 2.1. The Impact of COVID-19 on the HI and the Disease-Related Discussion: The Issues of Vulnerability and Resilience

Most of the studies gravitate towards disease-related discussions [16,17,18]. The emerging research field of HI and infectious diseases addresses the key concerns, ways of coping, business model innovation [19], risk perceptions [20], crisis management in extreme contexts [21], and the changes undertaken by the HI [22]. The impact of COVID-19 on the HI mirrors what occurred during previous pandemics such as SARS and MERS [23] and other disasters like the 2004 Indian Ocean Tsunami [24].

The effects of similar past events have been carefully considered to help frame the historical context [25,26] and understand and predict the consequences of the measures to fight the COVID-19 pandemic.

Baum et al. [27] highlight the immediate impacts of the current pandemic on the hospitality workforce, offering a critical assessment of the effects on the global hospitality workforce at three levels: macro (global, policy, government), meso (organisational), and micro (employee). At the macro level, the structural features of the HI generate precarity and vulnerability for hospitality workers. At the meso/organisational level, those companies that assign great attention to human resource management continue to improve their “people first” approaches [28] despite the uncertainty and look for creative ways to generate revenues, thereby employing people and ensuring a future for the business. In this vein, Reynolds et al. [29] consider that the HI employees have a role in ensuring the entertaining atmosphere and positively contributing to customer experience [30].

HI workers’ mood, motivation, and wellness have repercussions for providing service and health comprising physical, mental, and social well-being [31]. In this regard, prior studies on the tourism worker context have pointed out that long work hours and insufficient breaks are linked with reduced motivation and poorer customer service in the HI [32] and, more broadly, with increased turnover, especially in young hospitality and tourism employees [33]. In a similar vein, we claim that the young people who want to access the HI and work in this sector face many challenges due to the current pandemic. COVID-19 generated mobility restrictions at the microlevel that have negatively affected people’s capacity to participate in hospitality and tourism activities [34].

A vulnerability assessment based on the perceptions of practitioners (including business leaders among cruise lines, hotels, travel agencies, and touristic attractions) investigated through questionnaires and interviews depicts a grim picture in the short term, marked by economic loss, recovery concerns, and uncertainty. However, the prior studies did not investigate youth perceptions and their expected behaviours concerning precarity and vulnerability of future employment opportunities and careers in the HI.

The theory of resilience [35] has also been widely adopted before the pandemic as a conceptual framework including different dimensions (i.e., personal, community, economic, organisational, and systems resilience) in the HI literature. At the personal level, self-efficacy has proven instrumental in supporting one’s persistence in the face of aversive experiences and obstacles [36] affecting task effort, the level of goal difficulty chosen for performance, or expressed interest.

Adaptive capacity, flexibility, or fostering a culture which promotes innovation and self-efficacy, are critical factors in improving organisational resilience [37]. This capacity is strongly linked to the system’s overall vulnerability to a particular disaster or crisis event [38]. An integrative framework based upon six forms of capital (cultural, economic, human, natural, physical, and social capital) has been suggested as an extension of the theory of resilience, specifically geared towards developing disaster resilience in the hotel sector [37].

### 2.2. COVID-19 and Safety Compliance in the HI

To face the socioeconomic impacts on employees and one’s livelihood adjustments, hospitality operators and managers’ choices are primarily manifested through how day-to-day activities are undertaken and coupled with increasing awareness of new health and safety protocols [39].

COVID-19 has engendered a set of other health and safety regulations and procedures (e.g., social distancing) that are critical to closely monitor employee safety because protecting employees from the infection not only demonstrates the organisation’s responsibility to help contain the spread of the virus, but also determines the survival of the organisation during this crisis [40].

Organisations comply with the COVID-19 safety measures (requirements and protocols) to protect both employees and customers in response to this unprecedented health crisis and the consequent changes in working conditions [40]. Safety compliance refers to core safety tasks (including a set of behaviours that aim to meet an organisation’s safety requirements, i.e., safety rules and procedures, as well as wearing personal protective equipment) individuals carry out to maintain workplace safety [41]. Increased awareness of health and safety risks underpins the intention of attaining safety goals motivation that drives deep compliance behaviour beyond personal protection and other organisation’s members’ safety and incorporates a sense of moral responsibility for external stakeholders (i.e., customers) as well as the public [40].

Safety leadership has gained momentum during the current global crisis. It has been argued that hotel safety leadership positively affects employee safety behaviour (compliance, participation, and adaptation) [42]. Therefore, management strategies should emphasise safety leadership and promote employees’ safety behaviour from key aspects (i.e., safety coaching, care, motivation, and control), thus enhancing support of the organisation.

### 2.3. Employability and Job Insecurity in the HI

Job security is considered one of the essential parts of the quality of jobs. By contrast, job insecurity affects life and job satisfaction and commitment to the organisation [43,44].

Job insecurity refers to feeling concerned due to the perceived threat or uncertainty about the future of the job beyond the actual protection of the employee’s rights [45,46]. Namely, subjective job insecurity—a personal concern about the future of the job—refers to the psychological dimension of job insecurity [47,48].

Prior studies have proved that perceptions of job insecurity depend on several causes and that the solutions adopted to cope with its negative effects depend on the following factors: type of the employment contract [46], self-rated job performance [44], as well as several employees’ attributes and individual/personal resources [49], such as age [50], trust [51], and support [52].

Job insecurity is also affected by the perception of employability which expresses the subjective evaluation of an employee’s ability to obtain a new job or maintain the existing one [53]. Çalıskan and Özkoç [54] empirically demonstrate that employees’ perceptions of job insecurity are positively affected by frequency and impact of change and negatively by planning involved in change; the perception of employability moderates the relationships between the characteristics of organisational change and job insecurity. The perception of employability has gained momentum in recent years due to increased concerns about job insecurity.

In this vein, it has been considered an essential source in helping employees to deal with changeable organisation environment [55,56]. However, both perception of employability and job insecurity studies have not received adequate attention in the tourism and hospitality literature, instead being considered critical features for current and potential employees in light of the pandemic that undermines job stability, especially for young people. Therefore, we postulated the following hypothesis:

**Hypothesis** **1** **(H1).** *The COVID-19 pandemic is a negative factor that initiates change in the HI, directly influencing the spirit of youth adventures (NFC > SofA)*.

### 2.4. Young People’s Perceptions of Work and Career in the Hospitality Industry

Investigating young people’s perceptions of work in the hospitality industry, Mooney [57] observed that although they form a high proportion of the hospitality workforce in many countries, young people are not adequately valued, and inequitable power relations persist in the HI. Their perceptions of work in the hospitality industry—as temporary and unpredictable rather than an aspirational career choice—are at the basis of high turnover. Therefore, it has been claimed that the HI should retain its youthful workers rather than wasting this finite human resource [58].

The results are consistent with previous research addressing four-year tourism management school students’ who reported unfavourable or negative evaluations towards several dimensions of working in the HI [59,60]. Hoque and Ashif [61] explored the students’ undergraduate tourism and hospitality perceptions in Bangladesh, and their results indicate that the students’ perceptions of careers in the HI are unfavourable because some factors, such as a friendly working environment, a safe job, excellent promotion prospects, and an attractive starting salary are not important in the HI.

Tuzunkan [62] examined the perceptions and attitudes of undergraduate Korean tourism students. On the one hand, the findings showed that a tourism job is interesting, worth doing, needs fewer skills, people can use their ability and skills and get pleasure while working. On the other hand, some negative perceptions and prejudices about the tourist industry emerged.

Barron and Maxwell [63] pointed out that hospitality management students tended to have high ambitions for their future working careers when they began their studies. However, their ambitions changed after recognising the actual circumstances of the industry (negative working environments) and were disappointed with the HI when they had work experience. In addition, Khan and Krishanamurthy [64] revealed that factors like gender non-discrimination, promotion opportunities, and physical working conditions play a crucial role in motivating students, while other factors, such as the high risk of accidents, discourage the attitude towards choosing tourism as their future job and hence the tourism studies.

Knowledge, skills, and attitudes in specific areas (i.e., customer service, problem-solving, and leadership) are essential top competencies for hospitality managers and a priority concerning the educational process of a hospitality programme [65]. Interpersonal communication is a crucial skill for successful leaders in all organisations, including the service industry [66], while young people tend to be deficient in these skills when entering the business world [67].

Assuming the youth as a distinct workforce entity, Golubovskaya et al. [68] pointed out that hospitality jobs represent a critical developmental context for young people due to the misalignment with the workforce composition of the HI, the latter being dominated by young, often unexperienced workers. Therefore, they call for the need for recalibration and a pathway forward by paying greater attention to the development of talent and a more employee-focused and inclusive approach, allowing career jobs.

As a result of the literature review, we elaborated the following hypothesis:

**Hypothesis** **2** **(H2).** *The COVID-19 pandemic is a negative factor that initiates change in the HI and directly influences the attractiveness of jobs in this sector for young people (NFC > AofJ)*.

### 2.5. Job Attractiveness and the Spirit of Adventure in the HI

The theory of planned behaviour (TPB) argues that individual behaviour is influenced by attitudes, subjective norms, and perceived self-control [69]. Researchers have commonly used this theory to explain human behaviour in various studies. In this vein, some scholars argue that it represents a basic theoretical valuable framework in explaining individual behaviour in a pandemic context [70].

Uncertainty and inadequate knowledge decrease the perceived risk, which leads to lower control over practicing physical distancing and increasing intention to travel during the pandemic. Moreover, this theoretical lens has been used to investigate travellers’ destination choices of university students in China [70] and explain the consequences of COVID-19 for tourists’ behaviours, perceived travel risk, animosity, and intention to travel. Edwards et al. [71] consider that the work environment influences the attractiveness of a job, and the employees’ strategic renewal is positively affected by these factors and by demographic variables (i.e., age, gender, and educational level).

As per our knowledge, the studies aimed at evaluating the employment-generating capacity of the HI [72] did not deepen HI attractiveness concerning the youth. Young people’s behaviours in the HI have not received adequate attention, thus calling for further research to address young people’s perceptions and attitudes and contribute to filling this gap.

Kakoudakis et al. [73] investigated the socioeconomic benefits through job search behaviour, and their findings agree with those of Kanfer et al. [74] that the commitment to future employment is one of the main factors that can influence people when they choose a HI job. The self-determination theory and commitment offer interesting insights into what sustains human behaviour in work situations [75].

Based on the literature review, we suggested the following hypothesis:

**Hypothesis** **3** **(H3).** *The spirit of youth adventure developed for a job in the HI directly influences the attractiveness of jobs in this sector for young people (SofA > AofJ)*.

The individual’s inspiration for engaging in inherently interesting and enjoyable actions is nourished by the desire to satisfy the basic psychological needs mentioned above. When individuals evaluate certain activities as contributing to satisfying these needs, they are prone to continue performing those activities.

Self-motivation or autonomous motivation is essential for active engagement, involvement, and persistence in activities. As the highest form of autonomous motivation, intrinsic motivation is associated with autonomy and competence needs [76]. The need for relatedness—the desire to feel connected to others—is satisfied when people experience close relations (colleagues, clients, friends, and the family).

The workplace provides individuals with opportunities for social contacts and networks that contribute to satisfying this need. However, the contribution of work toward relatedness may depend on the nature of work that can facilitate or limit interactions with others [76]. Emotional intelligence has been found to positively influence performance in various fields, including business and the HI, where employees were proved to perceive emotional labour when performing their direct line of work [77].

We consider that all employees in the HI can be actors of adventure games, and we argue that the spirit of adventure can inspire young people to choose a job in the HI. Therefore, we wanted to fill the literature gap related to the spirit of adventure and explain the positive impacts of adventure on the attractiveness of a job in the HI for young people. Prior literature explored the adventure related to an activity and not to an invisible enemy like the COVID-19 virus.

Starting from the definition of adventure given by Muller and Cleaver [78], “relatively high levels of sensory stimulation, usually achieved by including physical challenging experiential components”, we adapted it to a new framework generated by COVID-19. We defined the spirit of adventure of young people working in the HI as a challenge that motivates them to promote teamwork to deal with the complaints from clients and overcome the danger of COVID-19.

Based on the above literature analysis, we derived our hypotheses that are described as follows:

**Hypothesis** **4** **(H4).** *Evolution of the HI after the end of the COVID-19 period mediates the relationship between the spirit of youth adventure developed for a job in the HI and the attractiveness of jobs in this sector for young people (SofA > EHI > AofJ)*.

**Hypothesis** **5** **(H5).** *The COVID-19 pandemic is a negative factor that moderates the relationship between the evolution of the HI after the end of the COVID-19 period and the attractiveness of jobs in this sector for young people (AofJ > NFC > EHI)*.

Tourism researchers tend to approach the research problem from the perspectives of operators, the tourists’ perspective, and the perspectives of the residents of the destinations’ communities [79]. By contrast, little attention has been paid to the perspectives of future generations among the research streams that are gaining momentum (i.e., crisis and disaster management; safety management; tourism resilience).

**Figure 1:**
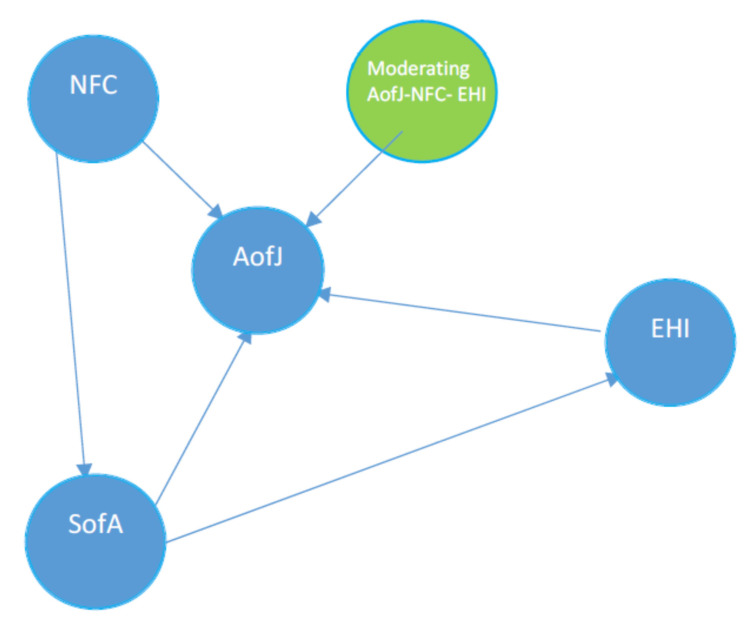
The theoretical model of the research. AofJ-the attractiveness of jobs in the hospitality industry for young people; EHI-evolution of the hospitality industry after the end of the COVID-19 period; NFC-the COVID-19 pandemic is a negative factor that initiates change in the hospitality industry; SofA-a job in the hospitality industry is motivated by the spirit of youth adventure.

To fill this gap, a conceptual model was formulated to illustrate the relationship between the attractiveness of jobs in the HI for young people, the evolution of the HI after the end of the COVID-19 period, the interaction between the people working in the HI in the COVID-19 pandemic scenario and the spirit of youth adventure (Figure 1).

## 3. Methodology

According to prior studies [34,61], the data used in our research were collected through quantitative survey-based research from 1 July to 31 October 2020.

The respondents were young Romanian people (121 employees from the HI, 75 students, and 112 unemployed people) who answered an online questionnaire. The questionnaire was pretested within a pilot study with a sample of 70 respondents over ten days.

We followed the example of Hair et al. [80] and the questionnaires with missing values were eliminated; at the end of the period, 308 valid questionnaires were obtained, of which 44.8% were answered by men, 55.2%—by women. Moreover, following Bagozzi and Yi [81], we checked the questionnaires for incomplete responses, and 25 responses were eliminated.

Concerning age, 211 respondents (68.5%) were 21–25 years old and 97 respondents (31.5%) were 26–30 years old. One of our research objectives was to validate the scale of measurement of the attractiveness of jobs in the HI for the youth during a crisis period. All the variables were measured using a five-point Likert scale from 1 (totally disagree) to 5 (totally agree). According to Chin et al. [82], we used the partial least squares structural equation modelling (PLS-SEM) approach to better explain the variance in the model’s dependent variables. PLS-SEM was used to clearly estimate formatively specified constructs and measure the model parameters [83,84].

After a confirmatory factor analysis, the items with loadings under 0.70 were removed, and Table 1 presents the final 14 items of the four variables that had their reliability values (outer loading) above the recommended value of 0.70 [85].

**Table 1:** Outer Loading and Variance Inflation Factors (VIF).

Variables/Items	Outer Loadings	VIF
AofJ—the attractiveness of jobs in the hospitality industry for young people—adapted from Yeh [86], who adapted from Gursoy and Gavcar [87]		
AofJ1—If I take protective measures and respect them, a job in the HI seems very attractive to me and will influence the development of high and new competences	0.845	2.533
AofJ2—I like working in the hospitality industry because I consider that team decisions are encouraged	0.878	2.673
AofJ3—I want to work in the hospitality industry because I can communicate with other people	0.845	2.465
AofJ4—I like working in the hospitality industry because I consider that personal risk has a low negative impact on my activities	0.847	2.443
AofJ5—I feel enthusiastic about working in the hospitality industry because I can put into practice my creative ideas	0.861	2.732
EHI—evolution of the hospitality industry after the end of the COVID-19 period—own scale		
EHI1—I consider that greater attention will be paid to human resources	0.860	1.902
EHI2—The hospitality industry will be reorganized according to the rules of social distancing because these rules will be maintained for a long time	0.885	2.304
EHI3—Communications between customers and employees will take place mainly through electronic devices	0.897	2.328
NFC—the COVID-19 pandemic is a negative factor that initiates change in the hospitality industry—own scale		
NFC1—The COVID-19 pandemic has positively affected the hospitality industry in terms of development of more sustainable and smarter offers and solutions	0.831	2.155
NFC2—The COVID-19 pandemic changed the vision about planned deadlines in the hospitality industry	0.899	1.930
NFC3—The COVID-19 pandemic improved the external regulatory demands for the hospitality industry	0.898	2.460
SofA—a job in the hospitality industry is motivated by the spirit of youth adventure—own scale		
SofA1—As a possible employee in the hospitality industry, I consider that the COVID-19 virus is a challenge that I will overcome	0.788	1.626
SofA2—Teamwork becomes a challenge because it turns into a win–win–win gamble: I protect myself—I protect you—we protect clients	0.873	1.993
SofA3—To deal with complaints from clients is a challenge for me in a pandemic crisis such as this generated by COVID-19	0.883	1.801

Analysing Table 1, we observe that the highest outer loading value (0.899) is registered by the items of the variable related to the COVID-19 pandemic as a negative factor that initiates change in the HI.

An item of the variable related to the spirit of youth adventure (0.788) allows us to affirm that the COVID-19 pandemic differently influencing the perception of adventure by young people registers the lowest outer loading value. This pandemic crisis has improved the external regulatory demands for the HI (0.898) and influenced the development of high and new competences (0.845).

Please see the detailed analysis of the values of the outer loadings in Section 4.

In Table 1, variance inflation factor (VIF) values of latent variables fall between 1.626 and 2.732, which takes cognisance of those issues related to collinearity. We continued measuring the reliability of the internal scale through Cronbach’s alpha, Dijkstra–Henseler statistics (rho_A), composite reliability (CR), and the average variance extracted (AVE) methods. The Cronbach’s alpha value ranges from 0.809 to 1.000 and proves the constructs’ consistency reliability [88].

The values of rho_A are above 0.7 (0.840 to 1.000) and are in consensus with the previous studies of Schuberth et al. [89] who showed that rho_A is a coefficient that requires values greater than 0.6. The composite reliability ranges between 0.833 and 0.936, and the CR is greater than 0.6 according to Bagozzi and Yi [81] or 0.7 according to [80]. All the five AVE values are above 0.50. Thus, we proved that the results are reliable, and the internal consistency and convergent validity are good. Moreover, we analysed the discriminant validity through the heterotrait–monotrait ratio (HTMT) and observed that HTMT values are not greater than 0.90 [90] and prove that the convergent validity is good.

We tested the structural model using R squared to measure the levels of the variables, and these values are moderate and range from 0.214 to 0.618. The structural model was also assessed using the standardized root-mean-square residual (SRMR), and the value of this indicator is 0.078; it does not surpass the threshold value of 0.10, confirming adequate goodness of model fit [91].

## 4. Findings and Discussions

A breakdown of the hypotheses testing is presented in Table 2.

The first hypothesis is validated (*p* = 0.000 and T-value = 6.508). It underlines the mediating role of the HI’s evolution after the COVID-19 period between the spirit of youth adventure developed for a job in the HI and the attractiveness of jobs in this sector for young people (SofA > EHI > AofJ). Thus, when the evolution is positive, the spirit of adventure is emphasised, and the attractiveness of jobs in the HI increases.

The second hypothesis is validated, and it proves that the COVID-19 pandemic is a negative factor that moderates the relationship between the HI’s evolution after the COVID-19 period and the attractiveness of jobs in this sector for young people (*p* = 0.002 and T-value = 6.762).

The third hypothesis shows that the spirit of youth adventure developed for a job in the HI directly influences the attractiveness of jobs in this sector for young people (*p* = 0.000 and T-value = 4.208), and it is validated.

The fourth hypothesis is also validated and shows that the COVID-19 pandemic is a negative factor that initiates change in this sector and has a psychological effect on young people and influences the attractiveness of jobs in this sector of activity (*p* = 0.003 and T-value = 2.984).

The fifth hypothesis proves that the COVID-19 pandemic is a negative factor that initiates change in the HI and the interactivity between people working in the HI in the COVID-19 pandemic scenario (*p* = 0.000 and T-value = 10.030), and it is validated. Table 3 shows path coefficient values and the specific indirect effects.

Age (*p* = 0.201) and gender (*p* = 0.199) do not influence the attractiveness of jobs in the HI for young people. These findings prove gender equality and an equal opportunity for young people to have a job in the HI. It is obvious that the spirit of youth adventure mediates two relationships: first, between the changes initiated in the HI and the attractiveness of jobs in the HI (*p* = 0.000), and second, between the changes-initiated change in the HI and its evolution after the COVID-19 period (*p* = 0.000).

The work environment [72] influences the attractiveness of a job. Therefore, we oriented our research to young people with the potential to evaluate the attractiveness of a job in the HI from other perspectives and transform the challenges of the COVID-19 pandemic into opportunities, for example, the opportunity to turn teamwork into a win–win–win gamble: I protect myself—I protect you—we protect clients—and contribute to the development of high and new competences of the young people in this sector.

Most young people consider pandemics a challenge to overcome, and they will choose to work in the HI regardless of the risks. However, some young people do not like working under the pressure generated by the COVID-19 pandemic and consider that the risk of becoming infected with COVID-19 is a challenge for them as they measure their level of protection. Our findings are sustained by Rather’s [92] research that concluded that perceived risk had a significant negative effect on Indian people during the COVID-19 crisis. These findings prove that young people will not avoid a job in the HI because it offers them the possibility of combining challenges with their adventure spirit.

Houge Mackenzie and Raymond [93] identified the key elements of adventure in New Zealand guide experiences related to determinants of people’s psychological well-being, and one of them was preventing ill-being. Consequently, during a pandemic crisis, the spirit of adventure of young people is moderated by self-protection and self-health. Young people consider that only high quality of services will make the difference between HI companies, and they are very confident that a new vision and their adventure spirit will concur to the improvement of the quality of services in the HI [94].

Ntounis et al. [95] arrived at the conclusion that in England, the people working in the HI are affected by the lack of information about the lockdown’s duration, and for this reason, when they return to a new normality, they will need additional ongoing assistance.

As a result, the pandemic crisis does not only negatively influence touristic service quality because it contributes to going ahead, thus soliciting the development of more sustainable and more thoughtful offers and solutions, changes the vision about planned deadlines in this sector, and highly improves the external regulatory demands for the HI [21]. Moreover, young people consider communication face-to-face with other people (i.e., colleagues and customers) as an opportunity to develop their competences and overcome the barriers raised by electronic devices. On the one hand, research on the HI and perceptions under extreme conditions has mainly investigated other categories’ perceptions, such as operators’ perceptions or a travellers’ or residents’ perception towards the existing and future tourism development [85].

We proved that the HI is fascinating for young people that could develop their competences and satisfy their spirit of adventure. Thus, the HI needs to rebuild and reimagine jobs amid the coronavirus crisis to reduce the level of COVID-19 exposure [96].

Finally, the findings point out that young people manifest a high spirit of adventure and want to work in the HI because the personal risk has a low negative impact on their activities, and if they take protective measures and respect them, a job in this sector seems very attractive to them.

Consequently, we consider that employment risk in the HI is associated with the spirit of adventure for young people. The spirit of adventure can be related to managing stress, and this strange relationship can be explained by this critical component of the HI represented by the COVID-19 pandemic. Paradoxically, these two factors can be transformed in motivation for young people if the hospitality environment offers them the image of well-being and unexplored adventures.

Our findings agree with the research of Skurvydas et al. [97] that recommended the future strategies be focused on product adjustment and transformation of business structures, seeking governmental guidance and policies in restoring market confidence in the HI.

## 5. Conclusions

The COVID-19 crisis poses considerable risks to education and employment [98]. Governments are forced to adjust plans to cover both public services (e.g., education) and economic sectors (e.g., tourism) to build back better for all generations. This objective will be attended by integrating a stronger focus on young people and future generations in government action and letting the youth and intergenerational considerations be mainstreamed in the governments’ strategies for the economic response to COVID-19. These actions will include measures to help the youth develop their skills, gain professional experience, and contribute to acting as connective tissue in public institutions and decision-making processes [99].

The crisis caused by coronavirus disease 2019 has posed wide-ranging problems for all sectors of the global economy. It has changed the world forever in an unimaginable respect and has heavily influenced international travel and tourist demands and the HI is tackling one of the most severe operational, commercial, and financial crises. All market players in the touristic and hospitality value chain areas have been forced to either minimize or completely stop provision of their services, resulting in the sudden and total cutoff of their revenue streams. Consequently, jobs lost or made redundant in the sector might take a long time to resurface and fully recover from the adverse impacts of the pandemic.

Our research’s originality consists of analyzing the relationship between the spirit, the adventure, and the attractiveness of jobs to young people in the HI. To the best of the authors’ knowledge, this study presents the first empirical evidence on young people’s attitudes and perceptions in this pandemic situation. Moreover, the significance of this study lies in the fact that its subject had not been addressed before from the perspective of the hospitality industry. It could be deciphered that the implications of our research are both theoretical and practical.

Theoretical implications. Due to the magnitude of the crisis and its devastating effects, previous conceptual and theoretical frameworks may prove inadequate or insufficient to understand its complex implications. Our research contributes to the tourism management literature by evaluating the impact of the pandemic on the future attractiveness of the HI to young people. Our theoretical research model underlines the importance of the spirit of youth adventure as a critical factor that generates new knowledge and develops young people’s work-related skills and general understanding of the job environment in the sector. The results relate to the HI system’s resilience from the socioeconomic perspective, affecting the macro-, meso-, and microlevels because of the impact on individuals, including the youth, businesses, communities, and nations [100].

Scientific discourse on the implications of the HI’s COVID-19 pandemic crisis framework is emerging. In this context, Cohen [101] emphasized environmental and social—rather than merely economic—concerns. In this regard, the future agenda should include a charter for sustainable tourism after the COVID-19 pandemic [102] that incorporates the enforcement of social distancing and regulations, safety measures and tools, stakeholder mapping, and anticipates changes in tourist behavior, ensures connectivity, and strengthens relationships.

The HI’s enthusiasm to return to normality is consistent with the youth’s spirit and orientation towards sustainability since the outbreak has revealed many changes in the relationships between sustainability in terms of intergenerational justice in the work environment and the HI. However, our study has proved that most young people have a strong desire to communicate face-to-face and be physically active in their workplaces. For this reason, these people prefer to work in the HI even in a pandemic crisis because they apply the rule: I protect myself—I protect you—we protect clients. The other contribution consists of validating the scale for evaluating the attractiveness of a job in the HI in a period of crisis. The validation of the scale is an answer to the recommendations by Gössling et al. [103] that outlined the potential negative impact of the COVID-19 crisis, suggesting that one of the HI’s main challenges is learning how to accelerate the transition to a sustainable future of the sector. Finally, we filled the gap in the HI literature related to the attractiveness of a job in the HI for young people during a period of crisis.

Practical implications. Our findings can ensure a better understanding of the managers and decisionmakers the HI to motivate young people to work in the sector that has been negatively impacted by this crisis. Young people consider that the HI will always be an opportunity for dreaming and keeping their souls young. Our study will be of interest to policymakers in terms of suggestions on how to transform challenges into opportunities by using the constraints imposed by a pandemic crisis that limits the socialisation between people, enhancing the creativity of young people working in the HI. The managers and decisionmakers in the HI should bear in mind that this current pandemic crisis is likely to reinforce this sector, and young people with a developed spirit of adventure represent a valuable workforce for their organizations because they are aware of clients’ expectations. They would certainly be ready to promote proactive communications. Our findings provide a practical value for managers and other decisionmakers, some motivational factors that would increase the attractiveness of their companies to young people, and research suggestions helpful to scholars involved in educational projects addressed to students of hospitality management programs to cultivate resilience capabilities by giving them inherent skills and competencies [104]. Finally, our research can help the managers and decisionmakers, particularly from the less developed countries, face social, economic, and environmental challenges and increase their organizational performance by using the young people’s spirit of adventure.

Regarding its limitations, we are aware that the findings from a study carried out in one European country are not likely to be wholly replicated elsewhere in the world. Therefore, we wanted to understand how these potential young corporate leaders perceive the impact of the pandemic on their future careers in the HI.

The future research will be oriented to improving the young people’s relationship with their spirit of adventure and responsibility. The new normality comes with a new approach to the activity in the HI and new challenges for both employees and employers. In this new light, sustainability acquires a new dimension that is much more social and personalized according to the HI’s particularities and spirit of adventure of the employees in the HI. The business model in the HI will be delineated by the pandemic crisis on the one hand and the new normality on the other.

## Figures and Tables

**Table 2:** The breakdown of the hypotheses testing.

	Original Sample (O)	Sample Mean (M)	Standard Deviation (STDEV)	T-Statistics (|O/STDEV|)	*p*-Values	Hypotheses
SofA > EHI > AofJ	0.267	0.267	0.041	6.508	0.000	H1: supported
Moderating AofJ–NFC–EHI	0.430	0.426	0.064	6.762	0.000	H2: supported
SofA > AofJ	0.245	0.248	0.058	4.208	0.000	H3: supported
NFC > AofJ	−0.181	−0.181	0.061	2.984	0.003	H4: supported
NFC > SofA	0.462	0.463	0.046	10.030	0.000	H5: supported

**Table 3:** Path coefficients and specific indirect effects.

	Original Sample (O)	Sample Mean (M)	Standard Deviation (STDEV)	T-Statistics (|O/STDEV|)	*p*-Values
Age > AofJ	0.041	0.041	0.032	1.279	0.201
Gender > AofJ	−0.046	−0.045	0.036	1.287	0.199
NFC > SofA > AofJ	0.113	0.114	0.026	4.379	0.000
NFC > SofA > EHI	0.271	0.272	0.032	8.325	0.000

## Data Availability

The data presented in this study are available on request from the corresponding author.
